# Optical Density and Polarized Light Microscopy to confirm calcification of Intra-ocular Lens

**DOI:** 10.22336/rjo.2024.60

**Published:** 2024

**Authors:** Avadhesh Oli, Simran Dhami

**Affiliations:** Head Vitreoretina and Uvea Services, Post Graduate Department of Ophthalmology, Command Hospital Air Force Agaram Post Bangalore, India

**Keywords:** calcified IOL, optical density, calcium deposits over IOL, polarized light, IOL, IOL = Intraocular Lens, UBM = Ultrasound biomicroscopy, OD = Optical Density

## Abstract

**Introduction:**

Intra-ocular lens (IOL) calcification is a rare yet serious complication, presenting as clouding within the optics of IOL and necessitating IOL exchange.

**Patient and Clinical Findings:**

In this case, a 77-year-old male experienced gradual vision loss in his left eye (LE) over four years post-cataract surgery a decade before. His best corrected visual acuity (BCVA) was 3/60 in the LE, with the anterior segment displaying a clear cornea but an opacified IOL within the capsular bag. Ultrasound biomicroscopy (UBM) revealed hyperechoic reflections from the IOL in the LE. ImageJ software used on UBM images indicated a significant difference in optical density (OD) compared to the right eye (RE).

**Results:**

The patient was diagnosed with IOL calcification in LE, and underwent anterior vitrectomy and IOL exchange with sulcus-placed IOL with optic capture in the capsular bag, resulting in an improved visual acuity of 6/18. Microscopic examination of explanted IOL revealed whitish calcium carbonate crystal deposits on the IOL, which were visible under polarized light.

**Conclusions and significance:**

The innovative use of polarized light and ImageJ software can be used in resource-constraint settings to confirm the diagnosis in such cases. Based on ImageJ on the UBM image, OD can pick up even subtle changes in the optical clarity of the IOL optic preoperatively.

## Introduction

IOL calcification is an infrequent yet noteworthy complication following cataract surgery. This phenomenon is predominantly associated with foldable hydrophilic acrylic IOLs, commonly called hydrogel lenses [1]. Three distinct categories of calcification have been identified: primary, secondary, and false positive calcification [2]. Lab diagnosis methods involve different techniques such as direct microscopy, histological examination with special stains like alizarin red and von Kossa stain, and chemical analysis using Scanning Electron Microscopy and Energy-dispersive X-ray spectroscopy [3].

IOL calcification incidence has reduced over the years due to constant improvement in the design and biomaterial used for making the IOLs [4].

The clinical examination reveals calcification in most of the cases, however, in certain situations, the findings could be subtle. The innovative use of optical density using ImageJ software [5] on the ultrasound biomicroscopic images compared to the fellow eye as described in the report can aid in subtle cases. The calcification can be further confirmed using polarized light, which can be useful in resource-constrained settings.

### *Patient Consent Statement* Informed written consent was obtained from the patient for publication of the case

## Case report

**Fig. 1:**
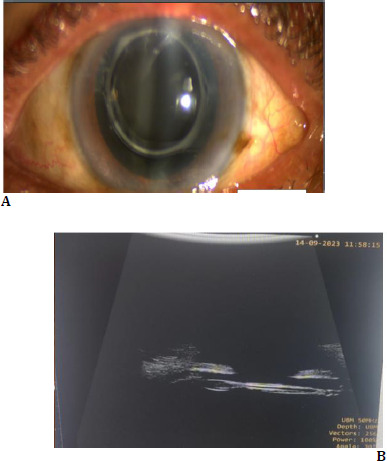
**A**. Slit lamp photograph with calcified IOL; **B**. UBM with calcified IOL

**Fig. 2:**
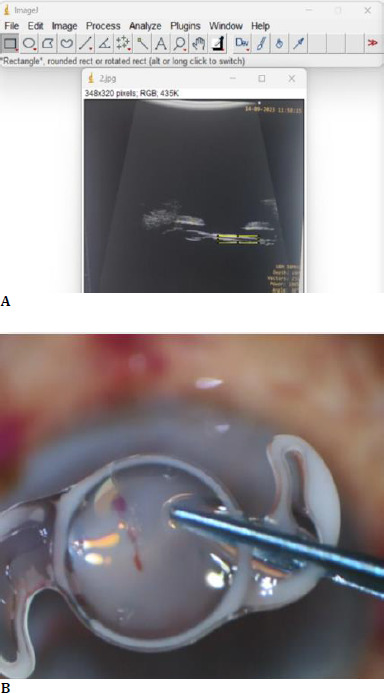
**A**. UBM image for optical density in ImageJ software; **B**. Calcium deposits on explanted IOL

A 77-year-old male reported painless progressive vision loss in his LE over four years, ten years post-cataract surgery. He denied any history of pain, redness, watering, flashes, or floaters. Upon examination, the best corrected Visual Acuity (BCVA) in the RE was 6/9 and LE was 3/60. Anterior segment evaluation in LE revealed a clear cornea but an opacified IOL within the capsular bag. Ultrasound Biomicroscopy (UBM) depicted hyperechoic reflections from the optic and haptics in the LE. The reflectivity from RE UBM images was less compared to the one of LE (**Fig. 1**). An innovative approach utilizing ImageJ software revealed a significant difference in optical density (OD) between the affected LE and the fellow RE. The OD was calculated using ImageJ software after converting the UBM image to 8 bits (**Fig. 2 A**). The method used for calculating the optical density is well described in the literature. ImageJ is open-code Java-based image-processing software (available at http://rsb.info.nih.gov/ij/index.html).

## Results

Given the severity of the condition and its impact on visual acuity, the patient underwent anterior vitrectomy and IOL explanation. The IOL was explanted by cutting the haptic to the center and pulling out by engaging the sharp edge in the 2.8 corneal incision (**Fig. 2 B**). Subsequently, a three-piece sulcus-placed Posterior Chamber IOL of power +21.5 D (Alcon Acrysof) was implanted with optic capture in the capsular bag. Limited anterior vitrectomy was done at 5000 cuts per minute and suction of 400 mmHg. The standard postoperative regimen was followed using Eye Drop Pred forte 1% 6 times daily with a gradual tapering dose. The final visual acuity improved to 6/18 at the end of six months. The IOP remained normal without evidence of other complications like uveitis, macular edema, or unusual inflammation.

**Fig. 3:**
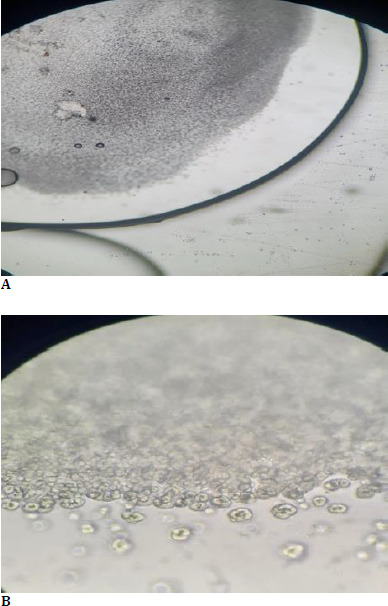
**A**. Microscopic examination of explanted calcified IOL; **B**. Polarized microscopy showing birefringence of calcium carbonate crystals

Microscopic examination of the explanted IOL unveiled whitish deposits on the optic and haptics, identified as calcium carbonate crystals (**Fig. 3 A**). Innovative polarized light microscopy further established these deposits’ confirmation, which provided a clear visualization of the calcific deposits on the IOL surface (**Fig. 3 B**).

## Discussion

In this case report, IOL calcification was diagnosed with innovative tools like ImageJ software preoperatively and polarized light microscopy postoperatively.

The intricacies of IOL calcification, its etiology, and the potential impact on visual outcomes are varied, based on the extent and location of calcification. Optical density measurement through ImageJ software and polarized light microscopy in confirming IOL calcification represents a novel approach to this rare complication diagnosis [5]. These tools aid in the accurate diagnosis and offer valuable insights into IOL calcification management, particularly in settings with limited resources.

The ImageJ software used for OD measurement in the presented case offered a quantitative assessment of the opacified IOL. This approach provided an understanding of the variations in optical properties between affected and unaffected eyes. The literature review further underscored the potential of optical density measurement as a reliable diagnostic tool in cases of variation in optical properties as in IOL calcification. It is important to note that accurate OD measurement with ImageJ requires proper image acquisition and pre-processing. Factors such as uneven lighting or artifacts in the image can lead to inaccurate measurements [6].

The innovative use of polarized light microscopy in calcium carbonate crystals confirmation on the IOL surface is another key aspect of this case report. The clarity and specificity offered by this technique in visualizing calcific deposits underscore its value in confirming the diagnosis of IOL calcification. This technique would be more useful in resource-constrained settings where specialized techniques like specific stains are unavailable [7].

The challenges associated with diagnosing IOL calcification are multifaceted. The rarity of the condition, coupled with its varied presentation, often makes it challenging to identify without specialized diagnostic tools. This report also highlighted the limitations of conventional diagnostic methods and the role of advanced techniques like optical density measurement and polarized light microscopy in overcoming these challenges.

The management of IOL calcification involves a multidisciplinary approach. According to the presented case, surgical intervention becomes imperative when the condition significantly affects visual acuity. The explantation of IOL from the fibrosed and calcified capsular bag remains a surgical challenge but a thorough gentle dissection using viscoelastic and silky hooks is advised [8].

The improvement in visual acuity from 3/60 to 6/18 following the surgical interventions underscores the significance of timely diagnosis and appropriate management in achieving favorable outcomes. Long-term prognosis and potential complications are also important aspects.

## Conclusion

In conclusion, IOL calcification, though rare, remains a significant post-cataract surgery complication with potential adverse effects on vision. This case report emphasizes the importance of employing innovative diagnostic techniques, such as optical density measurement and polarized light microscopy, to confirm the presence of calcification on the IOL surface. In resource-constrained settings, these tools can provide a cost-effective and efficient diagnosis, facilitating timely and appropriate management of this rare but impactful complication.

### What was known

IOL calcification can be diagnosed postoperatively using histopathological stains like 1% alizarin red, and von Kossa stain and using chemical analysis methods like Scanning Electron Microscopy, Energy-dispersive X-ray spectroscopy, all of which are difficult to access in resource constraint setting.

### What this paper adds

Innovative diagnostic techniques, such as optical density measurement and polarized light microscopy, that confirm the presence of calcification on the IOL surface have been described. In resource-constrained settings, these tools can provide a cost-effective and efficient diagnosis, facilitating timely and appropriate management of this rare but impactful complication.

### Future Directions

Further studies exploring the prevalence of this complication, refining diagnostic approaches, and evaluating the long-term outcomes of various management strategies can contribute to a deeper understanding of IOL calcification and enhance the quality of care provided to affected individuals.
